# Contextual variations in costs for a community health strategy implemented in rural, peri-urban and nomadic sites in Kenya

**DOI:** 10.1186/s12889-017-4140-z

**Published:** 2017-02-28

**Authors:** Charles Ouma Wafula, Nancy Edwards, Dan C. O. Kaseje

**Affiliations:** 1 The Tropical Institute of Community Health & Development, Great Lakes University of Kisumu, P.O. Box 2224-40100, Kisumu, Kenya; 2 School of Nursing, University of Ottawa, 1 Stewart Street, Room 205, Ottawa, ON Canada

**Keywords:** Community health strategy, Health, Costing, Contextual factors

## Abstract

**Background:**

Many low and middle income countries have developed community health strategies involving lay health workers, to complement and strengthen public health services. This study explores variations in costing parameters pertinent to deployment of community health volunteers across different contexts outlining considerations for costing program scale-up.

**Methods:**

The study used quasi experimental study design and employed both quantitative and qualitative methods to explore community health unit implementation activities and costs and compare costs across purposively selected sites that differed socially, economically and ecologically. Data were collected from November 2010 to December 2013 through key informant interviews and focus group discussions. We interviewed 16 key informants (eight District community health strategy focal persons, eight frontline field officers), and eight focus group discussions (four with community health volunteers and four with community health committee) and 560 sets of monthly cost data. Cost data were tabulated using Microsoft Excel. Qualitative data were transcribed and coded using a content analysis framework.

**Results:**

Four critical elements: attrition rates for community health volunteers, geography and population density, livelihood opportunity costs and benefits, and social opportunity benefits, drove cost variations across the three sites. Attrition rate was highest in peri-urban site where population is highly mobile and lowest in nomadic site. More households were covered by community health workers in the peri-urban area making per capita costs considerably less than in the nomadic settings where long distances had to be covered to reach sparsely distributed households. Livelihood opportunity costs for Community Health Volunteers were highest in nomadic setting, while peri-urban ones reported substantial employability benefits resulting from training. Social opportunity benefits were highest in rural site.

**Conclusions:**

Results show that costs of implementing community health strategy varied due to different area contextual factors in Kenya. This study identified four critical elements that drive cost variations: attrition rates for community health volunteers, geography and population density, livelihood opportunity costs and benefits, and social opportunity benefits. Health programme managers and policy-makers need to pay attention to details of contextual factors in costing for effective implementation of community health strategies.

**Electronic supplementary material:**

The online version of this article (doi:10.1186/s12889-017-4140-z) contains supplementary material, which is available to authorized users.

## Background

In an effort to deal with major gaps in health services delivery and growing health disparities, many low and middle income countries (LMICs) have developed community health strategies, which deploy lay community health volunteers (CHVs) to complement and strengthen core public health services [1, 2]. There is robust evidence of CHVs’ effectiveness [3–5] and some evidence of their efficiency [6]. However, many studies have only examined short-term impacts of CHV programs delivered on a limited, sub-national scale. Furthermore, many CHV programs have been supported in full or in part by external donors. If national governments are going to successfully move these CHV initiatives to scale and sustain implementation for health impact, costing considerations from the perspectives of both the government and society, and an understanding of cost variations across communities are paramount.

This paper describes considerations for costing the scale-up of CHVs based on results of a mixed methods study. The primary objectives of this study, undertaken in Kenya between 2009 and 2013, were to assess the uptake and effectiveness of the community health strategy; to evaluate the cost-effectiveness of this strategy; to describe the mechanisms and the perspectives of various stakeholders on task shifting; and to assess the quality of data collected by community health volunteers in different socio-demographic contexts. Primary study findings have been published elsewhere [7–11]. This article identifies variations in costing parameters pertinent to the deployment of CHVs across substantially different community sites and outlines considerations for costing program scale-up.

Systematic reviews of CHV effectiveness studies [1, 2, 12] report variations in impact that are influenced by populations served (e.g. rural versus urban), intervention intensity (e.g. health workers per capita, vertical versus integrated programs), delivery modalities (e.g. clinic, community meetings or mobile health technology), type of health professional trainers and supervisors involved (e.g. nurse, physician, midwife), and intervention components (e.g. training, supervision, referrals). All of these variations have potential cost implications as do program features such as the implementation phase (e.g. establishment versus maintenance phases); the mix of financing by service delivery partners (e.g. government, private sector and/or non-governmental organizations); and system governance and accountability [1, 13–15].

The foregoing factors are pertinent considerations for those making policy decisions about program scale-up. But in the literature on scale-up, cost parameters assessed have often involved simple estimates of coverage, such as basic arithmetic multipliers of population size [16]) or computing scale-up costs using only a few basic variations in context parameters (e.g. service delivery in rural versus urban settings) [5, 6]. While more recent modelling work on scale-up has increased the number of parameters under consideration [17], authors point to the limitations of empirical data to support the explication of relevant variables. Furthermore, there seems to be little agreement on underlying assumptions for estimating scale-up costs, making comparisons across studies difficult. For example, in a systematic review of thirteen cost-effectiveness modelling studies on pre-exposure prophylaxis for Human Immuno-Deficiency Virus (HIV) prevention [18], the authors noted considerable differences in costing-related assumptions that were made about the epidemic context (generalized versus concentrated epidemics), individual adherence levels, programme coverage for low-risk versus high-risk populations or prioritized intervention strategies. In a review of economic evaluations of non-communicable disease interventions in developing countries [19], authors described the inadequacy of valuing volunteer inputs; and, difficulty generalizing findings to other countries due to poor descriptions of context, including lack of details regarding “local costs, social concerns and political constraints” (p. 8).

Ministry of Health (MOH) perspectives are much more commonly represented than societal viewpoints in efficiency studies of community health programs involving CHVs [20]. But the functions of CHVs are undertaken in synchrony with the roles of government health workers. Thus, costing from both perspectives is essential to guide health system human resource planning, to gauge issues of recruiting and retaining CHVs and to determine whether or not CHVs can take on additional responsibilities [21].

Costing assumptions specific to the role of CHVs are also needed. CHVs have diverse characteristics including their motives for a position that is often voluntary [9]. CHVs work in a variety of settings and often outside of formal health facilities [1]. Incentive packages, needs for initial and ongoing training and supervision, and reporting and accountability relationships for CHVs both within the communities they serve and in the formal health care sector have cost implications for CHVs, their communities and the health system.

### Study setting and community health strategy

Kenya is a country with considerable variations in geography, culture, socioeconomic conditions and stability. Three areas of Kenya were purposively selected to reflect this variability. The three areas were rural, peri-urban and nomadic. The rural-agrarian site was situated in the Butere District of Western Kenya. The area is inhabited by a stable population with minimal in and out migration, mostly Christian, with strong social ties. The population density averages 81.8 per square kilometer [22]. The main source of livelihood is subsistence agriculture. The majority of the population lives within five kilometers of a health facility.

The peri-urban site was situated in Kisumu city, and is inhabited by an unstable population with high levels of in and out migration. It is mostly Christian, with weak social ties because of the mixed ethnic backgrounds typical of cosmopolitan informal urban settlements. The population density is extremely high, 225.4 per square kilometre [22] with casual labour and petty trade as the main sources of livelihood. The majority of the population lives within one kilometer of a health facility.

The nomadic site was situated in Garissa District of North-Eastern Kenya. The area is inhabited by a nomadic population living in harsh and dry climatic conditions. Hence the population moves seasonally in search of water and grass for their animals. The majority are Muslims, with strong social and religious ties. The population density is very sparse, 0.36 per square kilometer [22] with one uncertain rainy season. Their main source of livelihood is livestock. The majority of the population lives beyond five kilometers of a health facility.

A Community Health Strategy (CHS), launched in 2006 [23] aims to strengthen delivery of public health services. Under Kenya’s Public Health Act of 2009, these services include environmental health and food hygiene, curative services provided at health facilities, and promotive and preventive services provided in health facilities and via outreach [24]. The strategy aims to strengthen linkages between health providers and communities by establishing Community Health Units (CHUs). These units undertake regularized community dialogue jointly with health service providers for problem identification, planning, implementation of improvement actions through action days and monitoring and evaluation [7, 23]. CHVs form a critical component of the strategy and contribute to maintaining a competent, local, public health workforce [25].

Although the government aims to cover the entire country with the CHS, a lack of information regarding contextual variations in costs has been an impediment to effective national planning for scale-up. While some research has examined costs of the strategy, authors have not compared the costs across different contexts [11, 26].

## Methods

This cost analysis study was nested within a quasi-experimental study examining the uptake, implementation and effectiveness of the CHS [8, 10, 11]. The study employed both quantitative and qualitative methods to explore CHU implementation activities and compared costs across sites. This paper compares drivers of costs across sites from both governmental and societal perspectives.

The study sites included eight CHUs comprising four units in the rural location (Butere District), and two each in peri-urban and nomadic areas (Kisumu and Garissa, respectively).

Quantitative data was comprised of the financial costs incurred for activities. Costs were captured based on records maintained at four levels of CHS implementation; Community Health Unit, Community Health Extension Worker (CHEW), link Health Facility and the District Health Management level. Each of the four levels maintained predesigned activity logbook and expense and time record books that were used to record all cash and commodities received and payments and distributions made during each activity conducted. Expense and time records captured data on transport (fare and fuel paid), stationery, allowances (paid and or received), refreshment (lunch, teas, water, soft drink), accommodation costs (for residential meetings), telephone costs and equipment received (chalkboard, bicycle, motor cycle). On monthly basis, researchers reviewed and summarized quantities of commodities/equipment supplied, costs and time spent data from the activity logbook and expense and time record books. These data was keyed into computer using Excel package to allow for necessary tabulations of costs. Salaries for CHEWs were computed using MOH salary scales. The in-kind contributions of CHVs and Community Health Committee (CHCs) were tabulated based on the actual time they spent on CHS activities as registered by time logs. Cost was tabulated using rate of Kenya Shilling (KES) 287 per day, equivalent to farm clerk (based on education requirement and level of training of CHV and CHC as per the Regulation of Wages (Agricultural Industry) (Amendment) Order, 2011. Time spent by CHEWs, CHVs and CHCs during establishment and maintenance phases was captured using daily activity logbooks. The activities for which time was accrued for each phase are detailed below.

The establishment phase included activities related to: a) entry into the community to create awareness and joint planning for community health strategy establishment; b) participatory situation analysis leading to a community health strategy implementation plan; c) formation of a community health committee by electing and training members and linking them to the health system by representation on health facility committees; d) selecting and training community health volunteers to establish and maintain a community based health information system; e) level one health service delivery (including community mobilization, health education, counselling and referrals); f) supporting community health committees and community health workers to carry out complete registration of their households, analyzing this household data and summarizing it on community chalkboards, and; g) holding community dialogue sessions on the basis of analyzed household data, leading to action-oriented decisions that communities could then implement with the support of the health system.

The maintenance phase involved: a) regular household visits by community health workers, including up-dates of household registration, b) regular meetings of the community health committees and health facility committees at which community based information system data were presented and discussed to inform decisions and action; c) regular updates of and dialogue about information on chalkboards to encourage individual and community actions; and, d) periodic refresher training of community health workers and committees based on emerging skill gaps required for implementation.

At each site, the government was providing and paying for core public health services and government staffs were in place to perform these services. The estimates discussed in this paper concern the incremental costs of the CHS, which complemented and augmented the base costs of core public health services. Cost data were collected for a period of 1 year and 2 months and the attrition rates of CHVs were assessed for 3 years.

Costs for establishing a CHU were collected during the 2 month period required to form and launch the unit. Cost data for the subsequent maintenance period were collected on a monthly basis, over a period of 12 months. Five types of questionnaires and checklists were used to capture the cost data. Tool 1 was a District Health Management tool covering cost items related to training and supervision and meetings for CHS; tool 2 was a health facility tool that collected data on meetings arising from the CHS requirements; tool 3 collected cost data on activities of CHEW; tool 4 a CHC tool capturing cost data of meetings, dialogue and action days, transport and logistics, household registration, data analysis, chalkboard maintenance, allowance and stationery; and tool 5 was a CHV tool capturing costs of meetings, trainings, household registration, data analysis, chalkboard maintenance, household visits, home-based care, allowance, stationery, and referrals.

Costs of these activities were calculated in KES using prevailing prices during year 2011. The prevailing exchange rate as at 31st December 2011 was used to convert currency from KES into United States dollars (US $). This exchange rate was KES 84.8 per 1 US $ (Central Bank of Kenya). Costs are presented in US $.

Approval for use of cost data was granted by the Principal Investigator for the main study, which examined the uptake, implementation and effectiveness of the CHS.

Qualitative methods involved key informant interviews (*n* = 16) with the four District Health Management Teams of the study sites, to identify activities and other inputs required by these teams, Health Facility Management Committees, the CHEW, CHCs and CHVs to implement their community health strategy responsibilities. Eight focus group discussions were held with CHVs (*n* = 4 groups) and community health committees (*n* = 4 groups), and eight key informant interviews were conducted with CHEW to explore reasons for CHV attrition. Additional files show interview guides in more detail [see Additional files 1, 2, 3 and 4].

Records of CHVs from the beginning of each year were maintained in each site, taking note of CHVs who left and those who joined in replacement. Attrition of CHVs was classified into natural and forced attrition. Natural attrition was defined as leaving the community volunteer service by their own volition while forced attrition consisted of those who left due to Ministry of Health changes in the CHV guidelines [27]. The latter stipulated a change in CHV allocations to be in accordance with population density and type of settlement.

The nomadic site experienced a 1 year lag compared to the other sites in commencing CHV recruitment. Replacement CHVs were equally trained through routine activity based trainings, which was done for all CHVs before any activity would be undertaken.

An analysis of cost data from each phase was supplemented by the field experience of research leads (DK, CW) in each of the study areas and findings from an analysis of qualitative data [9] collected from CHVs in these settings. Using the Ministry of Health guideline on community health strategy implementation [23], activities cost data were tabulated using Microsoft Excel sheet so as to obtain costs required for implementing each activity.

Attrition rates were defined as the annual drop-out rate of CHVs. Annual drop-out rates were tabulated based on the number of CHVs who started at the beginning of the year but left before year end. To obtain average attrition rates for the period covered, 3 year-end periods (2011, 2012 and 2013) were used for rural Butere; 2 year-ends (2012 and 2013) were used for nomadic Garissa; and 2 year-ends (2011 and 2012) were used for peri-urban Kisumu as shown in Table 2. CHVs recruited to replace those who left were not included in computed attrition rates.

Data on how socio-economic factors affected CHV attrition were collected using qualitative methods, focus group discussions and key informant interviews. Qualitative data from the focus group discussions and key informant interviews were transcribed, anonymized, and coded using a content analysis framework as described by Miles and Huberman [28]. Transcripts were read and reread to identify reasons CHVs left or continued in their role. Comparisons were then made across study sites to identify different cost drivers across the three study areas.

## Results

Average per capita annual implementation financial costs for the community health strategy, from the Ministry of Health viewpoint, were highest at US$ 3.6 for rural CHUs followed closely by the nomadic at US$ 3.2 compared with US$ 0.5 for peri-urban CHUs (see Table 1).

**Table 1:** Per capita financial cost

Site	CHU average area (km^2^)	CHU average pop (2009 census)	Average annual cost/CHU ($)	Average # of CHVs/CHU	Per CHV			Pop. per capita cost (US $)
					Area	Pop.	Cost	
Rural	8.4	5592	20,354	36	0.2	153	558.6	3.6
Peri-urban	4.0	30,350	15,472	66	0.1	463	236.2	0.5
Nomadic	157.8	4377	14,135	15	10.5	292	942.3	3.2

*CHU* community health unit, *CHV* community health volunteer, 2009 census = Kenyan national census figures

### Cost drivers

Four critical elements appeared to drive cost variations across the three sites: population density, attrition rates for CHVs, livelihood opportunity costs and benefits, and social opportunity benefits.

### Population density

Population density is the number of individuals in a given square kilometer (km^2^). As shown in Table 1, peri-urban areas were the most dense, with a population of 30,350 people in an area of 4.0 km^2^, and the nomadic region the least dense with a population of 4377 people in an area of 157.8 km^2^. The rural site had 5592 people in an area of 8.4 km^2^. A CHV in a nomadic site covered the largest area (10.5 km^2^) compared to a counterpart in the peri- urban (0.1 km^2^) and rural (0.2 km^2^) sites. However, a CHV in a peri-urban CHU cared for over three times the number of people on average (463 people) compared to a CHV in a rural site (153 people), and almost twice that of a CHV in a nomadic site (292 people). CHVs in nomadic sites had long distances to cover for community training, household visits and dialogue sessions as compared to their counterparts in peri-urban and rural sites. Opportunity costs of the program for government employees were also more substantial in nomadic areas than either rural or peri-urban areas because of the substantial time required for this travel.

### Attrition rates

Attrition concerns how soon CHVs become inactive in their role following training. Average annual average natural attrition rates were highest (33%) in the peri-urban area followed by rural and nomadic areas (15% and 9%, respectively). See Table 2. Both the peri-urban and nomadic sites recorded a reduction in natural attrition rates (from 35 to 31% and 18 to 0% respectively) in their second year of study, while the rural site’s natural attrition rate increased from 13 to 22% in this period.

**Table 2:** Attrition rate

Year	Rural (Butere)			Nomadic (Garissa)		Peri-urban (Kisumu)	
	# of CHVs by yr end	% of attribution		# of CHVs by yr end	% of attribution	# of CHVs by yr end	% of attribution
		Forced	Natural		Natural		Natural
2010	193		Base yr	-	-	187	Base yr
2011	167		(26)13%	34	Base yr	122	(65)35%
2012	104	(26)16%	(37)22%	28	(6)18%	84	(38)31%
2013	93		(11)11%	28	(0)0%	-	-
Average			15%		9%		33%

Calculations for average attrition rates for both nomadic and peri-urban sites were based on 2 years, while those for rural on 3 year. Replacement CHVs were not included in the computation for attrition

A younger group of individuals were trained as CHVs in the peri-urban area than in the other two settings. CHVs reported that their training provided opportunities for job mobility; consequently, some left the area to pursue other employment opportunities. CHVs in the peri-urban area reported that the training provided opportunities for recognition and employment by non-governmental organizations (NGOs) and other community based organizations working in the area; this was described as the main reason for attrition. CHVs in the rural site reported increased opportunities for recognition by local politicians and government officers. This positioned them well for short-term, task-based wages, paid for assignments and employment by local administrators within the area. Another key reason for attrition in the rural setting was moving to urban areas either to join husbands working in cities or to search for paying jobs.

Implementation of the Ministry of Health revised guidelines [27] on CHV distribution by population density caused a forced attrition of 16% of CHVs in the rural site between 2011 and 2012. This site recorded the highest attrition rate across sites during the year in question. The revised guidelines aligned household coverage to CHVs by population density increasing CHV household coverage from an average of 250 to 500 people for areas with dense populations in the provinces of Nairobi, Central, Western and Nyanza provinces, and harmonizing a CHV to cover 50 people in sparsely populated areas in North Eastern province. Neither the peri-urban nor the nomadic site experienced forced attrition because they had CHV numbers already lower than stipulated in revised guidelines.

### Livelihood opportunity costs and benefits

Livelihoods encompass people’s capabilities, assets, income and activities required to secure the necessities of life [29]. In the nomadic area, poverty was extreme and livelihoods precarious. When lay health workers committed time for CHV activities, this significantly cut into their work as livestock keepers and potentially put their families at risk due to time spent away from farming. This created a substantial opportunity cost that was exacerbated because these CHVs indicated that they received little or no in-kind compensation for their volunteer services from the community. In the Nomadic site, these factors produced an expectation of being paid for time spent on CHV training and other community activities [9] that was not directly realized. However, qualitative interviews showed that opportunities for further training and recognition by politicians and NGOs working in the area increased for the CHVs in the nomadic sites. These opportunities increased CHV's likelihood of being included by other organizations in paid-for-tasks such as community mobilization and logistic support. For instance a CHV from the Nomadic site reported as follows:

*“We (CHVs) are the people that politicians use whenever they want to reach the communities in our areas, and also NGOs that come to these areas use us to make them reach the people including helping to distribute commodities”*

Peri-urban CHVs reported little in-kind compensation from the community. However, the CHV training provided some with a means to pursue other opportunities and formal training programs, defraying the social burden of underlying opportunity costs associated with CHV training and service provision. A CHV from the peri-urban site noted as follows:

*“Some of our members have been selected by MOH and NGOs for further skills training leading to their being employed and for many of us this is a motivation”*

Compensation for rural CHVs from the community was reported as reasonable. This compensation occurred due to social recognition of their contributions, an increased opportunity for paid-for tasks by Ministry of Health and other Non-governmental Organizations working in the area and a perceived advantage for longer-term formal employment as local administrators and/or local political leaders. A rural CHV observed:

*“.........and so far we have had at least two of our members being successfully interviewed and hired as local administrators and one as a member of County Assembly”*

### Social opportunity benefits

This concerns the relationship between being a CHV and one’s social connectedness in the community. In both rural and nomadic regions, CHVs were working in communities where they had grown up and were known. In the rural setting, participants described an improvement in their social status when they completed training and took on the role of a CHV. Their CHV work also enhanced their social connectedness. In the nomadic setting, where populations were sparse, the catchment areas for service delivery did not necessarily align with traditional social ties, thus extending the period that a CHV needed to be engaged to realize social benefits from the CHV role. For instance, a CHV said:

*“Initially they were disrespectful towards us and they were making some utterances like, − ‘Where are you from, what do you want and why are you just filling in these forms and then leaving, and why do you ask these questions?’ But we had had the community dialogue and they have been educated and they now understand what we are doing and they are still advocating that we should have a policy that makes it easy for them to identify us. All in all they currently know us and love us and our service to the community as well.”*

However, in the peri-urban setting, high patterns of migration particularly among youth meant that their role as a CHV was not necessarily appreciated or recognized by the community. While CHV training did not appear to elevate or extend their social capital among the population served, it did increase social connections with health providers in the formal health care system. Qualitative interviews showed that social connectedness with health providers in informal health care system appeared to be a common benefit across the sites. In some cases, this provided an entry point to pursue a higher level of education.

## Discussion

The parameters identified from this study could be used as a basis for comparative costing assumptions and sensitivity analyses relevant to the establishment, maintenance and scale-up of CHV programs in diverse contexts. Prior work on CHV effectiveness studies have highlighted variations in impact that are influenced by populations served, intervention intensity, delivery modalities, type of professional provider involved, and intervention components. Our study extends this work and reveals four critical elements of cost variations in the implementation of community health strategy. Two elements - CHV attrition rates and per capita coverage by CHVs - have been described as pertinent considerations for scale-up by others [30] both in terms of health resource planning for the formal government system and volunteer community health services. Our study demonstrates the need to differentiate between forced and natural attrition. Our study also indicates that CHS implementers need to pay closer attention to how and why attrition rates of CHVs vary across different social, economic, and ecological contexts. In our study, the peri-urban site had the highest natural attrition rate followed by the rural agrarian and nomadic sites. Furthermore, the stage of program implementation matters for the attrition of CHVs. For instance, attrition in the first year was higher than in later years, across all sites mainly due to CHVs joining with higher expectations (of direct income such as allowances and wages), who moved on quickly when those expectations were not met. Attrition rate then slowed in the second year, suggesting that the remaining CHVs were more likely ready to work under the prevailing conditions of the job as CHVs. Cost wise, this attrition trend indicated that increased training needs and costs have to be catered for, considering extent to which replacements will remain needed. Longer term studies are needed across different contexts to better understand natural attrition rates and to develop better assumptions for sensitivity analyses so these can be taken into account in costing programs for scale-up. The population density of the area appeared to have implications for opportunity costs and benefits. The opportunity costs appeared higher in nomadic sites as compared with rural agrarian and peri-urban sites, due to long distances to be covered by both the CHVs and government staff supporting implementation of the strategy. For instance, variances were observed among sites with respect to how much time was need for routine weekly meetings, household visits and community dialogue and action days. A CHV working in the nomadic site required one full day for routine weekly meetings or to attend community dialogue and action days, as compared with a CHV in either a rural or peri-urban site who needed at most, a half day for the same tasks. Time spent on the strategy related activities in the field was also higher for ministry of health staff in the nomadic site compared to peri-urban or rural sites. Similarly, costs of both transport and time spent by the respective cadres were comparatively higher in nomadic sites as compared with peri-urban sites and rural sites.

This study identified two other key cost considerations that have received little attention: livelihood opportunity costs and benefits, and social opportunity benefits. These considerations are especially important for costing models that include a societal viewpoint [15]. However, they are also important for estimating costs from a Ministry of Health perspective, since they are likely to have a direct impact on the retention of CHVs and government costs for recruitment, training, and supervisory activities. While these two additional costing considerations are consistent with theories on social capital and social connectedness [31, 32], quantifying these so they can be used in a sensitivity analysis remains an area for further development. Altering the relative ranking of these factors across settings might be useful in sensitivity analyses and would improve the estimates used in tools such as the Marginal Budgeting for Bottlenecks Toolkit (MBB) developed by United Nations Children's Fund (UNICEF) and The World Bank – Version September 7, 2007 [33].

Proxy measures may have some utility for these additional factors. For example, the socioeconomic level of a particular catchment area indirectly manifests the livelihood opportunity costs and benefits that might be accrued. However, as illustrated by our findings, it may not adequately capture more subtle social influences and social ties, which impact on monetary or social rewards and incentives for CHV work. Likewise, this proxy does not capture the nature of personal and social risk being assumed or social benefits being accrued by individuals and their communities with the implementation or maintenance of a CHV program.

There were hints of some other sources of cost variation that arose during the field work conducted as part of this study. For instance, stakeholders described their experience of health programs having been initiated and/or supported by external donors (which is the reality for many LMICs). Communities may have different expectations of incentives and payment when a non-governmental organization or other external donor is involved. The CHV program examined in this study was financed by the Ministry of Health rather than by external donors. However, the community expectations (particularly in the nomadic and peri-urban settings), reflected prior experience with external donors providing health services. A situation analysis and discussions with decision-makers during study implementation suggested that the nomadic region appeared to have the highest level of dependency, with community members expecting to be lifted out of their poverty circumstances without necessarily making a community contribution to services provided. Initially, this made it more difficult to recruit CHVs at that site. It was the reason for the 1-year delay in study implementation s well as the low numbers of CHUs and CHVs established and recruited. A very precarious livelihood under conditions of extreme poverty, famine and war renders communities more prone to expectations that someone coming from outside will pay for all program costs incurred. This factor has the potential to influence not only the actual cost of a community health strategy, but also the acceptability of who pays.

There are additional variations in cost that we did not systematically capture, such as those resulting from the use of different types of personnel in core programs (e.g. nurses, midwives, other health professionals); attrition, absenteeism and redeployment rates among core health services personnel; and related consequences for effective supervisory supports. Some of these have been identified previously [34]. Variations in core services and how these may interact with the cost parameters identified in this study require further consideration.

Another issue requiring further investigation is the variation in service opportunity costs for MOH personnel associated with implementing a CHV program. These programs are dependent on government health personnel who recruit, train and supervise CHVs. While in the medium to long term, the presence of a CHV is intended to increase the reach and effectiveness of the services provided, in the short term, other activities of the government-employed health professionals may be foregone, such as health education, patient follow up, and assisting expectant women to develop and use individual birth plans and first level of care, as they recruit, train and supervise CHVs.

Using institutional economics as the theoretical basis for their analysis, some authors have identified institutional factors and governance within communities as an area that might be useful to consider in further examinations of contextual variations that drive costs [6, 35]. Alternative theoretical frameworks for costing analyses are critical to advance this field. Such an analytic framework would have been useful to apply, a priori, for this study.

Sustainability is another facet of programs that may be linked to intervention costs. This Kenyan study looked at costs of an earlier phase of program sustainability (denoted as the maintenance phase, with 2 to 3 years of follow up after the initial training period). Although this is one of the longer periods of CHV follow up described in the literature [36, 37], one might anticipate further costing considerations to arise over a longer period. More consistent documentation of attrition rates over time and across contexts would build a stronger body of knowledge on what influences these rates and how this affects short versus long term costs. From the Ministry of Health perspective, other inflationary costs might need to be considered in planning for longer-term sustainability. Similarly, from a societal perspective, levels of community dependency on external donors for subsidies and employment opportunities would be pertinent. Longer-term trajectories of social and livelihood opportunity costs are also needed. For instance, a targeted program on vaccination that demonstrates early visible impact on the health of communities may shift social opportunity costs and benefits for CHVs in a different way than programs with more diffuse and longer-term impacts.

## Conclusion

Affordable community health strategies in LMICs require the engagement of CHVs. Community health strategy has the potential for scale-up to increase access to and coverage of basic health services. However, health programme managers and policy-makers need to pay close attention to the details of contextual factors in implementation of the strategy. Through a comparison of the costs and cost drivers in three substantially different community sites, we have highlighted considerations for costing programs of community health strategy scale-up in health service delivery.

## Additional files

Additional file 1: Key informant Interview guide for District Community Health Strategy Focal Person (DCHSFP). (DOCX 13 kb)
Additional file 2: Key informant Interview guide for Community Health Extension Worker (CHEW). (DOCX 12 kb)
Additional file 3: Focus group discussion guide for Community Health Committee members (CHC). (DOCX 13 kb)
Additional file 4: Focus group discussion guide for Community Health Volunteers (CHVs). (DOCX 12 kb)

## Abbreviations

CHC: Community health committee
CHEW: Community Health Extension Worker
CHS: Community Health Strategy
CHU: Community Health Unit
CHV: Community health volunteer
HIV: Human immuno-deficiency virus
KES: Kenya Shilling
km^2^: Square kilometer
LMIC: Low and middle income countries
MBB: Marginal Budgeting for Bottlenecks Toolkit
MOH: Ministry of health
NGO: Non-governmental organizations
UNICEF: United Nations Children's Fund
US$: United States dollar
