# Supplementary material for: Contextual variations in costs for a community health strategy implemented in rural, peri-urban and nomadic sites in Kenya
Source: BMC Public Health. 2017 Feb 28;17:224. doi: 10.1186/s12889-017-4140-z (PMC5330022; doi:10.1186/s12889-017-4140-z)
Supplement: Additional file 2: — Key informant Interview guide for Community Health Extension Worker (CHEW). (DOCX 12 kb) [file 12889_2017_4140_MOESM2_ESM.docx]

**Additional file 2**

**GREAT LAKES UNIVERSITY OF KISUMU (GLUK)**

**Study: Contextual variations in costs for a community health strategy implemented in rural, peri-urban and nomadic sites in Kenya**

**KII Guide for Community Health Extension Worker (CHEW) TOOL KII/2**

As the link between Ministry of Health and Communities in this District ...........................(*insert name of District)*,

1) How has the government (and especially the ministry of health) facilitated your effective implementation of community health strategy activities? ***(probe for training, sensitization, resourcing, equipment and commodity supply, stationery material, transport logistics, among others)***

2) By identifying specific input (the what) that government provided towards implementation of community health strategy activities, comment on adequateness to facilitate your compliance with CHS implementation guidelines of 2007 in this District. ***(probe through phases of establishment, maintenance and sustainability; for training budget, CHV and CHC training, motivation budget and sustainability, funding of CHS community activities, equipment and commodity supply, stationery material, transport logistics, among others)***

3) How does the size of area covered under your jurisdiction affect overall implementation of community health strategy activities? ***(probe on distance covered, number of people covered, work and sustainability of CHVs, life style of the people in the district, road network, communication network, on key activities of mobilizing community, selection and training CHVs and CHCs, supervision, meetings, etc)***

4) Comment on the role played by CHVs in implementation of CHS in this area. ***(probe on both positive and negative roles)***

5) In your opinion, what factors promote CHV retention in this area? **(probe for economic, social, health and capacity factors)**

6) In your opinion, what factors promote CHV attrition in this area? **(probe for economic, social, health and capacity factors)**

***.................................................................THANK YOU..................................................................***
