# Supplementary material for: Contextual variations in costs for a community health strategy implemented in rural, peri-urban and nomadic sites in Kenya
Source: BMC Public Health. 2017 Feb 28;17:224. doi: 10.1186/s12889-017-4140-z (PMC5330022; doi:10.1186/s12889-017-4140-z)
Supplement: Additional file 4: — Focus group discussion guide for Community Health Volunteers (CHVs). (DOCX 12 kb) [file 12889_2017_4140_MOESM4_ESM.docx]

**Additional file 4**

**GREAT LAKES UNIVERSITY OF KISUMU (GLUK)**

**Study: Contextual variations in costs for a community health strategy implemented in rural, peri-urban and nomadic sites in Kenya**

**FGD with Community Health Volunteers (CHVs) TOOL FGD/2**

As a critical workforce of this CHU...........................(*insert name of CHU)*,

1) How has the ministry of health facilitated your implementation of community health strategy activities? ***(probe for training, sensitization, resourcing, equipment and commodity supply, stationery material, transport logistics, among others)***

2) By identifying specific input that MOH provided towards implementation of community health strategy activities in your CHU, comment on adequateness to facilitate your compliance with CHS implementation guidelines of 2007 in this CHU. ***(probe through phases of establishment, maintenance and sustainability; for staff training budget, CHV and CHC training, motivation budget and sustainability, funding of CHS community activities, equipment and commodity supply, stationery material, transport logistics, among others)***

3) How does the size of area covered under your jurisdiction affect overall implementation of community health strategy activities? ***(probe on distance covered, number of people covered, work and sustainability of CHVs, life style of the people in the district, road network, communication network, on key activities of mobilizing community, selection and training CHVs and CHCs, supervision, meetings, etc)***

**4. Attrition:**

a) Explain for whom and why CHV work is attractive to, in this area.

b) Explain what benefits a CHV gets from their role and work ***(probe on both direct and in-kind benefits from MOH, Community, and other stakeholders).***

c) Explain reasons for attrition among CHVs ***(probe by; ages, sex, education level, etc).***

**5. Livelihood opportunity costs and benefits**

a) Explain how the work of a CHV impacts on their household livelihoods ***(probe on: time, income, etc).***

b) Comment on the adequateness of compensation that a CHV gets in return for sacrifices they make in volunteer work.

c) Explain any effects on identified economic livelihoods with regards to CHV retention and attrition in your CHU.

**6. Social opportunity benefits**

a) How is a CHV role perceived in this community where you work? Is the perception important to the CHVs themselves and why is it important? ***(Probe explanation for each view identified and seek for demonstrative testimonies).***

b) Explain any effect of identified social opportunity benefits on CHV retention and attrition in your CHU.

***.................................................................THANK YOU..................................................................***
